# Non-coding RNA-driven cardiovascular immunometabolic reprogramming: from inflammatory endotypes to therapeutic opportunities

**DOI:** 10.3389/fcell.2026.1882071

**Published:** 2026-07-22

**Authors:** Yang Sheng, Yao Yao, Hengcang Wang

**Affiliations:** 1 Department of Cardiology, Tongde Hospital of Zhejiang Province Affiliated to Zhejiang Chinese Medical University (College of Integrated Traditional Chinese and Western Medicine Clinical Medicine), Hangzhou, China; 2 Department of Pharmacy, Women’s Hospital, School of Medicine, Zhejiang University, Hangzhou, China; 3 Department of Internal Medicine of Traditional Chinese Medicine, Zhejiang Academy of Traditional Chinese Medicine, Hangzhou, China

**Keywords:** cardiovascular disease, extracellular vesicles, immunometabolic reprogramming, inflammatory endotypes, non-coding RNA, precision therapy

## Abstract

Cardiovascular disease is increasingly recognized as a heterogeneous immunometabolic disorder shaped by inflammation, metabolic rewiring, endothelial dysfunction, mitochondrial stress, and tissue remodeling across diverse cell types. This review provides a hypothesis-generating conceptual synthesis of non-coding RNA-driven cardiovascular immunometabolic reprogramming from an inflammatory endotype-oriented perspective. Because ncRNA profiling has not yet prospectively assigned cardiovascular patient cohorts to validated inflammatory endotypes, we frame mechanism-based endotypes as complementary research constructs rather than clinically deployable diagnostic categories. We discuss how conventional disease labels, including atherosclerosis, myocardial infarction, heart failure, hypertension, and cardiomyopathy, may be cross-mapped to dominant mechanisms such as athero-inflammation, sterile ischemic injury, fibro-inflammatory remodeling, metabolic inflammation, and vascular immune-endothelial dysfunction. We summarize how microRNAs, long non-coding RNAs, circular RNAs, and extracellular vesicle-associated RNA species regulate macrophage cholesterol handling, inflammasome activation, endothelial activation, vascular smooth muscle cell plasticity, cardiomyocyte mitochondrial dysfunction, fibroblast activation, extracellular matrix remodeling, and intercellular communication, with added attention to circRNA tissue-source patterns in cardiac, immune-cell, endothelial, and vascular compartments. We also highlight their context-dependent and cell type-specific actions, which challenge simple protective-versus-pathogenic classifications. Finally, we discuss translational opportunities, including circulating and extracellular vesicle-associated non-coding RNAs as liquid biopsy candidates, RNA-based therapeutics, endotype-enriched study designs, and traditional medicine-inspired multicomponent strategies. Despite barriers related to delivery, specificity, disease stage, species conservation, analytical standardization, and validation, integrated phenotyping, multi-omics profiling, functional perturbation, and biomarker-enriched trials may advance non-coding RNAs as candidate classifiers, regulators, markers, and therapeutic targets in precision cardiovascular medicine.

## 1 Introduction

Cardiovascular disease remains the leading cause of morbidity and mortality worldwide, but its biological complexity is still insufficiently captured by conventional classifications based on anatomy, hemodynamics, lipid burden, or ventricular function. Increasing evidence indicates that many cardiovascular disorders are driven not only by structural injury or metabolic risk factors, but also by persistent crosstalk among inflammation, immune-cell activation, metabolic rewiring, endothelial dysfunction, mitochondrial stress, and maladaptive tissue remodeling (Liang and Shimada, 2021; DeBerge et al., 2023). This shift has promoted the concept of cardiovascular immunometabolism, which views immune and metabolic pathways as interconnected regulatory networks that shape disease initiation, progression, and therapeutic response (DeBerge et al., 2023; Thorp and Karlstaedt, 2024). Within this framework, non-coding RNAs (ncRNAs) have emerged as key molecular regulators because they can coordinate gene-expression programs across immune cells, endothelial cells, vascular smooth muscle cells, cardiomyocytes, and fibroblasts (Fasolo et al., 2019; Sansonetti and De Windt, 2022).

**FIGURE 1:**
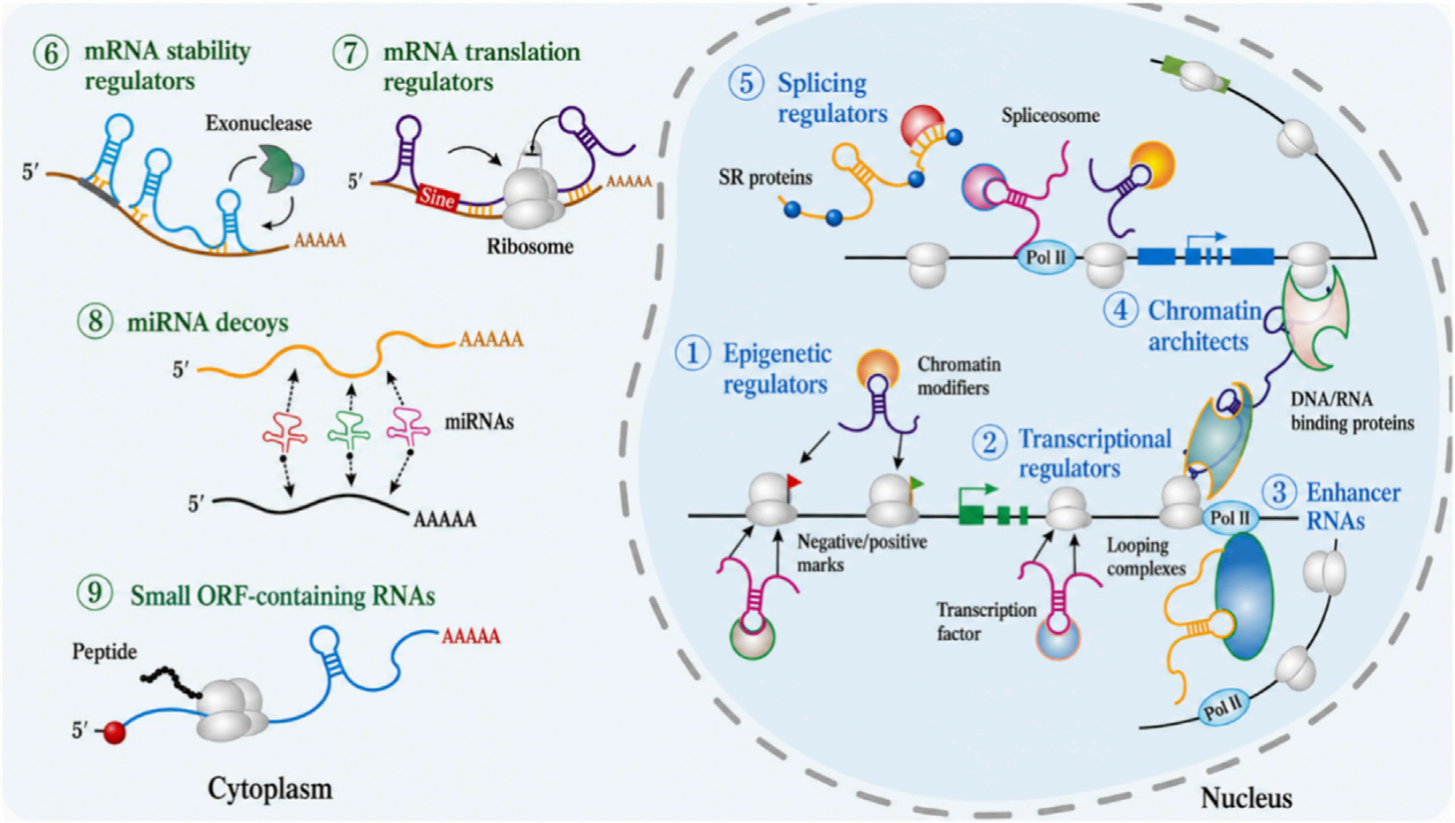
Regulatory modes of non-coding RNAs. Nuclear ncRNAs regulate chromatin state, transcription, enhancer activity, and splicing, whereas cytoplasmic ncRNAs control mRNA stability, translation, miRNA availability, and small ORF-derived peptide production. These mechanisms support multilayered ncRNA control of cardiovascular immunometabolic reprogramming.

NcRNAs, including microRNAs, long non-coding RNAs, circular RNAs, and extracellular vesicle-associated RNA species, are no longer considered transcriptional noise or passive disease markers. Instead, they participate in post-transcriptional repression, chromatin regulation, RNA sponging, transcript stabilization, intercellular communication, and stress-responsive signaling (Poller et al., 2018; Ardiana et al., 2023). In cardiovascular disease, these regulatory activities converge on macrophage cholesterol handling, endothelial activation, vascular smooth muscle cell plasticity, cardiomyocyte mitochondrial dysfunction, fibroblast activation, extracellular matrix remodeling, and inflammatory resolution (Schober et al., 2022; Caporali et al., 2024). Importantly, the same ncRNA may exert divergent effects depending on cell type, disease stage, metabolic context, and extracellular communication route, which explains why simple “protective versus pathogenic” interpretations are often inadequate (Nazari-Jahantigh et al., 2012; Li et al., 2016). As summarized in Figure 1, these activities span chromatin-level regulation, transcriptional and splicing control, and cytoplasmic regulation of mRNA stability, translation, miRNA availability, and small ORF-derived peptide production.

A major challenge in the field is that cardiovascular ncRNA studies have often been organized around broad clinical diagnoses, such as atherosclerosis, myocardial infarction, heart failure, hypertension, or cardiomyopathy. These categories are clinically useful but biologically heterogeneous. An endotype-oriented approach may provide a more precise framework by classifying patients according to dominant inflammatory and immunometabolic mechanisms, including athero-inflammation, sterile ischemic injury, fibro-inflammatory remodeling, metabolic inflammation, and vascular immune-endothelial dysfunction (Liang and Shimada, 2021; Genkel and Shaposhnik, 2020). Such a framework is particularly suitable for ncRNA research because ncRNAs can function both as mechanistic regulators and as circulating molecular signatures that reflect tissue injury, immune activation, metabolic stress, and extracellular vesicle-mediated communication (Poller et al., 2018; Viereck and Thum, 2017).

This review therefore examines ncRNA-driven cardiovascular immunometabolic reprogramming from three interconnected perspectives. First, it discusses inflammatory endotypes as a conceptual framework for ncRNA-based disease stratification. Second, it summarizes the mechanistic layers through which ncRNAs regulate immune, vascular, and cardiac resident-cell metabolism and inflammation. Third, it evaluates therapeutic opportunities, including ncRNA biomarkers, RNA-based interventions, endotype-guided treatment strategies, and multicomponent or traditional medicine-inspired approaches. By integrating mechanism, stratification, and therapeutic translation, this review aims to move cardiovascular ncRNA research beyond descriptive biomarker discovery toward precision immunometabolic intervention.

### 1.1 Narrative review methodology

We conducted a narrative literature review to synthesize mechanistic and translational evidence on ncRNA-driven cardiovascular immunometabolism. PubMed/MEDLINE, Web of Science, Scopus, and Google Scholar were searched for publications available from January 2000 to May 2026. Search terms included combinations of ‘non-coding RNA’, ‘microRNA’, ‘miRNA’, ‘long non-coding RNA’, ‘lncRNA’, ‘circular RNA’, ‘circRNA’, ‘extracellular vesicle RNA’, ‘cardiovascular disease’, ‘atherosclerosis’, ‘myocardial infarction’, ‘heart failure’, ‘hypertension’, ‘cardiomyopathy’, ‘immunometabolism’, ‘inflammation’, ‘macrophage’, ‘endothelial cell’, ‘vascular smooth muscle cell’, ‘fibroblast’, ‘mitochondria’, and ‘metabolic reprogramming’. Reference lists of key reviews and original articles were also screened manually.

Studies were included when they addressed cardiovascular ncRNAs in relation to inflammatory signaling, immune-cell activation, metabolic remodeling, endothelial dysfunction, extracellular vesicle communication, fibrosis, biomarker development, or RNA-based therapy. We prioritized mechanistic studies with functional perturbation, human cohort studies with cardiovascular relevance, clinical or translational studies, and recent high-quality reviews that clarified disease mechanisms or therapeutic barriers. Studies were excluded when they were not cardiovascular in focus, did not address ncRNAs, provided only descriptive expression changes without biological or translational context, or duplicated evidence already represented by more informative studies. Because this article is a narrative review rather than a systematic review or meta-analysis, examples were selected to illustrate reproducible mechanisms, endotype relevance, and translational feasibility rather than to exhaustively enumerate all reported ncRNAs.

### 1.2 How an inflammatory endotype framework advances cardiovascular ncRNA research

The main originality of this review is the use of inflammatory endotypes as an organizing framework for cardiovascular ncRNA biology. Most existing ncRNA reviews are structured by clinical disease labels, such as atherosclerosis, myocardial infarction, heart failure, hypertension, or cardiomyopathy. Such disease-based summaries are useful, but they often mix patients and models with different dominant mechanisms. No published cardiovascular cohort has yet demonstrated that an ncRNA panel alone can prospectively assign individuals to validated inflammatory endotypes with established thresholds. Therefore, the framework proposed here should be read as a hypothesis-generating research schema that links ncRNAs to disease-generating mechanisms, sample selection, cohort enrichment, and mechanism-matched intervention hypotheses, rather than as a clinically validated diagnostic algorithm. This framework also helps resolve apparent contradictions in the ncRNA literature. A transcript that appears pathogenic in macrophage-rich plaque inflammation may be protective during post-infarction repair, while an EV-associated RNA may function as a passive injury signal in one setting and an active intercellular mediator in another. Therefore, the proposed model goes beyond cataloguing ncRNAs by diagnosis and instead places each ncRNA within a cellular source, inflammatory context, metabolic state, disease stage, sample type, evidence level, and therapeutic implication. This perspective is intended to provide a conceptual bridge between descriptive ncRNA profiling and precision cardiovascular immunometabolic intervention.

## 2 Inflammatory endotypes in cardiovascular disease: a framework for ncRNA-Based stratification

Cardiovascular diseases are increasingly recognized as heterogeneous immunometabolic syndromes rather than uniform entities defined only by anatomy, hemodynamics, lipid burden, or ventricular function. The concept of inflammatory endotypes provides a useful framework for classifying patients according to dominant biological mechanisms, such as athero-inflammation, sterile ischemic injury, fibro-inflammatory remodeling, metabolic inflammation, or endothelial immune dysfunction. This framework is particularly relevant for non-coding RNA research because miRNAs, lncRNAs, circRNAs, and extracellular vesicle-associated ncRNAs can integrate inflammatory signaling, metabolic rewiring, and cell–cell communication across cardiovascular tissues. Instead of treating ncRNAs merely as isolated biomarkers, endotype-based stratification may help identify which ncRNA networks are mechanistically active in specific disease states and which patients are most likely to benefit from targeted immunometabolic intervention (Liang and Shimada, 2021; DeBerge et al., 2023; Genkel and Shaposhnik, 2020).

### 2.1 From conventional disease categories to inflammatory endotypes

Traditional cardiovascular classification relies heavily on clinical syndromes such as coronary artery disease, myocardial infarction, heart failure, hypertension, or cardiomyopathy, yet these labels often contain biologically diverse patient populations with different inflammatory and metabolic drivers (Liang and Shimada, 2021). The endotype concept shifts the focus from surface phenotype to disease-generating mechanisms, allowing cardiovascular disorders to be divided into mechanistically meaningful subgroups with distinct inflammatory, metabolic, genetic, and molecular features (Liang and Shimada, 2021; Genkel and Shaposhnik, 2020). This approach is especially valuable in heart failure, where similar clinical presentations may arise from ischemic injury, pressure overload, metabolic inflammation, immune activation, endothelial dysfunction, or fibrotic remodeling (Liang and Shimada, 2021; Francis et al., 2014). In atherosclerosis, endotype-oriented thinking is also useful because plaque progression may be dominated by lipid retention, monocyte–macrophage activation, inflammasome signaling, impaired cholesterol efflux, smooth muscle cell plasticity, or defective inflammation resolution (Schober et al., 2022; Genkel and Shaposhnik, 2020). Clinical evidence from anti-inflammatory cardiovascular trials further supports the need for mechanism-based stratification, because targeting interleukin-1β reduced recurrent cardiovascular events in patients with prior myocardial infarction and residual inflammatory risk, independent of lipid lowering (Ridker et al., 2017). However, the same evidence also highlights a critical limitation: broad anti-inflammatory therapy may reduce events in selected patients but does not fully define who has a specific inflammatory endotype, which inflammatory pathway dominates, or which molecular regulator should be targeted (Ridker et al., 2017; Ridker et al., 2020). This is where ncRNAs may provide added value, because they can capture upstream regulatory states that precede or accompany cytokine production, immune-cell activation, metabolic switching, and tissue remodeling (Fasolo et al., 2019; Sansonetti and De Windt, 2022).

### 2.2 Major inflammatory endotypes in cardiovascular disease

An athero-inflammatory endotype is characterized by lipid-driven innate immune activation, endothelial adhesion molecule expression, monocyte recruitment, foam cell formation, inflammasome activation, and plaque instability (Schober et al., 2022; Ridker et al., 2017). In this endotype, ncRNAs may act as regulators of macrophage lipid handling and inflammatory tone, as illustrated by miR-33-dependent control of cholesterol efflux and macrophage energy metabolism, and by miR-155-mediated regulation of macrophage inflammation and atherogenesis (Karunakaran et al., 2015; Horie et al., 2012; Du et al., 2014). LncRNAs also participate in this athero-inflammatory program, with CHROME regulating cholesterol homeostasis in primates and being elevated in plasma and atherosclerotic plaques from individuals with coronary artery disease (Hennessy et al., 2019). An ischemia–reperfusion sterile inflammatory endotype is driven by cardiomyocyte injury, mitochondrial dysfunction, danger-associated molecular pattern release, complement activation, neutrophil recruitment, macrophage transition, and delayed resolution of inflammation (DeBerge et al., 2023; Sansonetti and De Windt, 2022). In this context, ncRNAs may regulate the balance between injury amplification and repair by modulating immune-cell recruitment, cardiomyocyte survival, mitochondrial homeostasis, and cytokine signaling after myocardial injury (Sansonetti and De Windt, 2022; Kumarswamy and Thum, 2013). This endotype is important because excessive early inflammation can worsen tissue injury, whereas insufficient inflammatory resolution can impair scar maturation and promote adverse remodeling (DeBerge et al., 2023). A fibro-inflammatory remodeling endotype is defined by persistent immune activation, fibroblast expansion, extracellular matrix deposition, transforming growth factor-β signaling, and progressive ventricular stiffening or dilation (Sansonetti and De Windt, 2022; Kumarswamy and Thum, 2013). This endotype provides a strong rationale for studying ncRNA-mediated communication among immune cells, cardiomyocytes, endothelial cells, and cardiac fibroblasts, because many remodeling signals are transmitted through paracrine factors, extracellular vesicles, and non-coding transcripts (Sansonetti and De Windt, 2022; Poller et al., 2018). In heart failure, ncRNAs should therefore be interpreted not only as markers of cardiomyocyte stress but also as regulators of inflammatory persistence, fibrotic activation, and maladaptive repair (Sansonetti and De Windt, 2022). A metabolic-inflammatory endotype is particularly relevant to obesity, diabetes, metabolic syndrome, and heart failure with preserved ejection fraction, where systemic inflammation, insulin resistance, adipose tissue dysfunction, endothelial inflammation, and altered myocardial substrate utilization converge (Schiattarella et al., 2021; Paulus and Tschope, 2013). In HFpEF, obese-inflammatory phenotypes have been associated with differences in comorbidity burden, disease severity, and fibrosis, suggesting that metabolic inflammation is not merely a background risk factor but a disease-structuring mechanism (Sabbah et al., 2020). This endotype is highly suitable for ncRNA-based investigation because miRNAs and lncRNAs can regulate glucose metabolism, fatty acid oxidation, mitochondrial function, endothelial activation, and immune-cell metabolic polarization (DeBerge et al., 2023; Fasolo et al., 2019). A vascular immune-endothelial dysfunction endotype centers on endothelial activation, impaired nitric oxide bioavailability, oxidative stress, barrier dysfunction, leukocyte adhesion, and vascular smooth muscle cell phenotypic switching (Fasolo et al., 2019; Schober et al., 2022). Regulatory ncRNAs are particularly relevant here because they influence endothelial inflammation, smooth muscle cell plasticity, monocyte recruitment, and intercellular communication within the vascular wall (Schober et al., 2022; Wei et al., 2013). For example, miR-155 has been implicated in endothelial cells, macrophages, dendritic cells, and vascular smooth muscle cells during atherosclerosis, indicating that a single ncRNA may operate across multiple cellular compartments within the same inflammatory endotype (Ma et al., 2013; Zhu et al., 2011). As summarized in Table 1, cardiovascular inflammatory endotypes provide a practical framework for linking dominant pathogenic mechanisms with ncRNA-defined molecular classifiers and mechanism-matched therapeutic strategies.

**TABLE 1:** Inflammatory endotypes of cardiovascular disease and ncRNA-based stratification.

Inflammatory endotype	Core pathological features	Representative ncRNA-related mechanisms	Stratification or therapeutic implication	Refs
Athero-inflammatory endotype	Lipid retention, endothelial activation, monocyte recruitment, foam-cell formation, inflammasome activation, and plaque instability	miR-33 and CHROME regulate cholesterol handling; miR-155 modulates macrophage inflammation	May identify patients with residual inflammatory risk and guide targeting of macrophage lipid metabolism or plaque inflammation	Schober et al. (2022), Karunakaran et al. (2015), Hennessy et al. (2019)
Sterile ischemic injury endotype	Cardiomyocyte injury, mitochondrial dysfunction, DAMP release, neutrophil infiltration, macrophage transition, and delayed resolution	miR-210 regulates hypoxia response and mitochondrial metabolism; EV-enriched miR-27a-5p/miR-27a* is linked to MI-related/chronic HF remodeling and antioxidant-defense signaling	Useful for distinguishing injury-amplifying inflammation from reparative inflammation after myocardial infarction	DeBerge et al. (2023), Chan et al. (2009), Tian et al. (2020)
Fibro-inflammatory remodeling endotype	Persistent inflammation, fibroblast activation, TGF-β signaling, extracellular matrix deposition, and ventricular remodeling	miR-21, miR-29, and Wisper regulate fibroblast activation, ECM remodeling, and cardiac fibrosis	Supports anti-fibrotic ncRNA strategies and biomarker-guided monitoring of adverse remodeling	van Rooij et al. (2008), Micheletti et al. (2017), Thum et al. (2008)
Metabolic-inflammatory endotype	Obesity, diabetes, insulin resistance, adipose inflammation, mitochondrial dysfunction, and altered myocardial substrate use	Metabolism-associated miRNAs regulate lipid handling, mitochondrial function, and immune-cell metabolic polarization	May help stratify patients with metabolic syndrome, diabetes, or HFpEF-related inflammatory cardiovascular disease	Thorp and Karlstaedt (2024), Schiattarella et al. (2021), Sabbah et al. (2020)
Vascular immune-endothelial dysfunction endotype	Endothelial activation, oxidative stress, impaired nitric oxide signaling, leukocyte adhesion, and VSMC phenotypic switching	miR-126 regulates endothelial repair; miR-92a controls angiogenesis; miR-145 maintains VSMC phenotype	May guide assessment of endothelial injury, vascular repair capacity, and plaque remodeling risk	Schober et al. (2014), Bonauer et al. (2009), Cordes et al. (2009)

### 2.3 ncRNA signatures as molecular classifiers of inflammatory endotypes

Circulating ncRNAs may help classify inflammatory endotypes because they can be detected in plasma, serum, whole blood, or extracellular vesicles and may reflect disease activity across vascular, myocardial, immune, and metabolic compartments ( Sansonetti and De Windt, 2022; Poller et al., 2018). However, current evidence supports these transcripts mainly as candidate classifier layers rather than validated clinical stratification tools. Compared with single cytokines or conventional biomarkers, ncRNA panels may capture broader regulatory programs, including immune-cell activation, metabolic rewiring, endothelial dysfunction, and fibrotic remodeling (Fasolo et al., 2019; Schober et al., 2022). For instance, a patient with coronary artery disease and high hsCRP may show residual inflammatory risk, but ncRNA profiling would still need to be integrated with plaque imaging, inflammatory cytokines, lipid traits, cell-source attribution, and outcome data before a true athero-inflammatory endotype assignment could be made (Schober et al., 2022; Ridker et al., 2017). Similarly, in heart failure, ncRNA signatures may help generate hypotheses about ischemic inflammatory remodeling, metabolic-inflammatory HFpEF, immune-cell-driven cardiac inflammation, and fibro-inflammatory progression, but they do not yet replace ejection fraction, imaging, natriuretic peptides, fibrosis markers, or clinical phenotyping (Liang and Shimada, 2021; Sansonetti and De Windt, 2022). A major challenge is that many reported ncRNA biomarkers are derived from small cohorts, heterogeneous sample types, different detection platforms, and insufficient external validation (Poller et al., 2018). Another challenge is biological interpretability, since altered ncRNA abundance in blood does not automatically prove causal involvement in a specific cardiovascular cell type or inflammatory pathway (Fasolo et al., 2019). Therefore, future studies should integrate circulating ncRNA profiles with single-cell transcriptomics, spatial mapping, metabolomics, proteomics, imaging biomarkers, and clinical inflammatory markers to build more reliable endotype models (Liang and Shimada, 2021; DeBerge et al., 2023).

### 2.4 Critical perspective

The inflammatory endotype framework is conceptually attractive, but its value depends on whether it can improve causal understanding, risk prediction, and therapeutic decision-making beyond existing clinical categories (Liang and Shimada, 2021; Genkel and Shaposhnik, 2020). For ncRNA research, the key question is not simply whether a transcript is upregulated or downregulated in cardiovascular disease, but whether it defines a reproducible mechanism-linked subgroup and can guide intervention (Fasolo et al., 2019; Schober et al., 2022). A rigorous endotype-oriented ncRNA strategy should distinguish correlation from causality, circulating signals from tissue-specific regulation, and disease-associated biomarkers from druggable molecular nodes (Sansonetti and De Windt, 2022; Poller et al., 2018). If these challenges are addressed, ncRNA-based endotyping may provide a mechanistic bridge between cardiovascular immunometabolism and precision therapy, enabling more selective targeting of athero-inflammation, sterile myocardial inflammation, fibro-inflammatory remodeling, metabolic inflammation, and endothelial immune dysfunction (Liang and Shimada, 2021; DeBerge et al., 2023).

### 2.5 Translating endotypes into a conceptual ncRNA stratification summary

To avoid implying current clinical readiness, Table 2 is presented as a conceptual summary rather than a practical diagnostic tool. It summarizes how dominant mechanisms can be connected with candidate ncRNAs, biospecimen choices, evidence status, therapeutic implications, and current limitations. Evidence status was assigned using explicit criteria: human tissue or circulating association, cell-source attribution, functional perturbation, animal or large-animal validation, independent cohort validation, and clinical trial evidence. Strong mechanistic/human support denotes human association plus reproducible functional evidence; moderate support denotes consistent association with incomplete causal or external validation; early clinical translation denotes available first-in-human or trial data without validated endotype-based clinical use.

**TABLE 2:** Conceptual summary of candidate ncRNA signatures across cardiovascular inflammatory endotypes.

Endotype	Dominant mechanism and non-ncRNA features	Candidate ncRNAs and likely source	Evidence status	Interpretation and limitations
Athero-inflammatory (Schober et al., 2022; Karunakaran et al., 2015; Hennessy et al., 2019; Holdt et al., 2016)	Lipid retention, endothelial activation, monocyte recruitment, foam-cell formation, NLRP3/IL-1beta/IL-6 signaling, plaque necrotic core, and plaque instability	miR-33, miR-34a, CHROME, MeXis; miR-155 only as a context-dependent macrophage/EV signal; circANRIL as a 9p21 vascular/macrophage-related circRNA	Strong mechanistic and human tissue/circulation support, but no validated endotype threshold	Supports residual inflammatory-risk hypotheses; miR-155 targeting requires cell type, stimulus, and disease-stage qualification
Sterile ischemic injury (DeBerge et al., 2023; Chan et al., 2009; Song et al., 2022; Tian et al., 2020; Vausort et al., 2016)	Acute cardiomyocyte necrosis, DAMP release, mitochondrial ROS, neutrophil influx, monocyte-macrophage transition, and resolution failure	miR-210, miR-92a, miR-27a-5p/miR-27a*, EV-ncRNAs; cardiac or ischemia-linked circSLC8A1, circNFIX, and MICRA	Strong preclinical and early human biomarker support; limited time-resolved validation	Needs temporal sampling to distinguish passive injury release from active RNA-mediated signaling
Fibro-inflammatory remodeling (Micheletti et al., 2017; Hinkel et al., 2020; Taubel et al., 2021; Tikhomirov et al., 2026; Boichenko et al., 2025)	Persistent cytokine and IL-11/TGF-beta signaling, macrophage-fibroblast communication, myofibroblast expansion, ECM deposition, LV dilation or stiffness	miR-21, miR-29, Wisper, miR-132/CDR132L, IL-11-linked miR-27b-5p and miR-497-5p; macrophage circHIPK2 after MI	Strong mechanistic support and early clinical pharmacodynamic data; phase 2 efficacy not confirmed for miR-132 inhibition	Requires fibrosis imaging, biomarkers, and stage-specific selection; fibroblast-selective delivery remains unresolved
Metabolic-inflammatory (Thorp and Karlstaedt, 2024; Schiattarella et al., 2021; Sabbah et al., 2020; Boichenko et al., 2025)	Obesity, diabetes, insulin resistance, adipose inflammation, endothelial inflammation, mitochondrial dysfunction, and HFpEF comorbidity clusters	miR-33, miR-34a, miR-210, MALAT1 in metabolic-stress contexts; circulating fibrosis/severity ncRNAs	Moderate support; many studies are associative or comorbidity-confounded	Interpret with BMI, diabetes, renal function, sex, age, medication exposure, inflammatory markers, and metabolomic features
Vascular immune-endothelial dysfunction (Schober et al., 2014; Bonauer et al., 2009; Cordes et al., 2009; Michalik et al., 2014; Boeckel et al., 2015; Cao et al., 2018)	Endothelial activation, NO impairment, oxidative stress, leukocyte adhesion, barrier dysfunction, and VSMC phenotypic switching	miR-126-5p, miR-92a, miR-145, MALAT1 in endothelial/hematopoietic compartments; endothelial circRNAs cZNF292 and circHIPK3; EV-associated miRNAs	Strong vascular-cell and endothelial-EV evidence with variable external clinical validation	Not automatically stronger than athero-inflammatory evidence; main limitations are cell-source attribution and vascular-bed specificity

### 2.6 Proposed assignment criteria for cardiovascular inflammatory endotypes

Patient or tissue assignment should not rely on ncRNA expression alone. In principle, an inflammatory endotype assignment would require concordance among clinical context, conventional biomarkers, imaging or histology, immune and metabolic readouts, and a candidate ncRNA panel. At present, no universally accepted biomarker thresholds define these categories; the criteria below are therefore proposed as research-grade operational definitions for cohort enrichment and hypothesis testing rather than as clinical diagnostic rules. The proposed research-grade assignment criteria are summarized in Table 3.

**TABLE 3:** Research-grade criteria proposed for assigning cardiovascular inflammatory endotypes.

Endotype	Typical clinical or tissue context	Non-ncRNA molecular/imaging features	Candidate ncRNA layer	Current assignment status
Athero-inflammatory (Schober et al., 2022; Ridker et al., 2017; Holdt et al., 2016)	CAD, ACS, plaque tissue, or residual inflammatory risk despite lipid lowering	hsCRP, IL-6, IL-1beta/NLRP3 activity, plaque macrophages, necrotic core, FDG-PET or CCTA plaque features	miR-33, context-qualified miR-155, CHROME, MeXis, circANRIL	No validated threshold; use multimodal enrichment
Sterile ischemic injury (DeBerge et al., 2023; Song et al., 2022; Vausort et al., 2016)	Acute MI, ischemia-reperfusion injury, or infarct-border tissue	Troponin/CK-MB, DAMPs, mitochondrial ROS, neutrophil-to-lymphocyte ratio, CMR edema or infarct size	miR-210, miR-92a, EV-miR-27a, circSLC8A1, circNFIX, MICRA	Time-dependent; sampling window is essential
Fibro-inflammatory remodeling (Micheletti et al., 2017; Tikhomirov et al., 2026; Boichenko et al., 2025)	Post-MI remodeling, HFrEF, pressure overload, aortic stenosis, or chronic remodeling tissue	NT-proBNP, galectin-3, sST2, PICP/PIIINP, CMR ECV/LGE, COL1A1/COL3A1/ACTA2, IL-11/TGF-beta signaling	miR-21, miR-29, Wisper, miR-132/CDR132L target engagement, miR-27b-5p, miR-497-5p, circHIPK2	Research-grade; requires fibrosis phenotype and disease-stage definition
Metabolic-inflammatory (Schiattarella et al., 2021; Sabbah et al., 2020; Boichenko et al., 2025)	Obesity, diabetes, metabolic syndrome, HFpEF, or adipose-cardiac inflammatory stress	HbA1c/HOMA-IR, adipokines, CRP/IL-6, mitochondrial/metabolomic markers, microvascular dysfunction	miR-33, miR-34a, miR-210, MALAT1, circulating HF fibrosis/severity ncRNAs	Comorbidity-adjusted models are needed
Vascular immune-endothelial dysfunction (Schober et al., 2014; Boeckel et al., 2015; Cao et al., 2018)	Hypertension, diabetes, CAD-related endothelial dysfunction, or vascular-bed-specific injury	Impaired FMD/NO, VCAM-1/ICAM-1/E-selectin, oxidative stress, endothelial EVs, VSMC switching markers	miR-126-5p, miR-92a, miR-145, MALAT1, cZNF292, circHIPK3	Vascular-bed and cell-source specificity remain unresolved

## 3 Mechanistic layers of ncRNA-driven cardiovascular immunometabolic reprogramming

**FIGURE 2:**
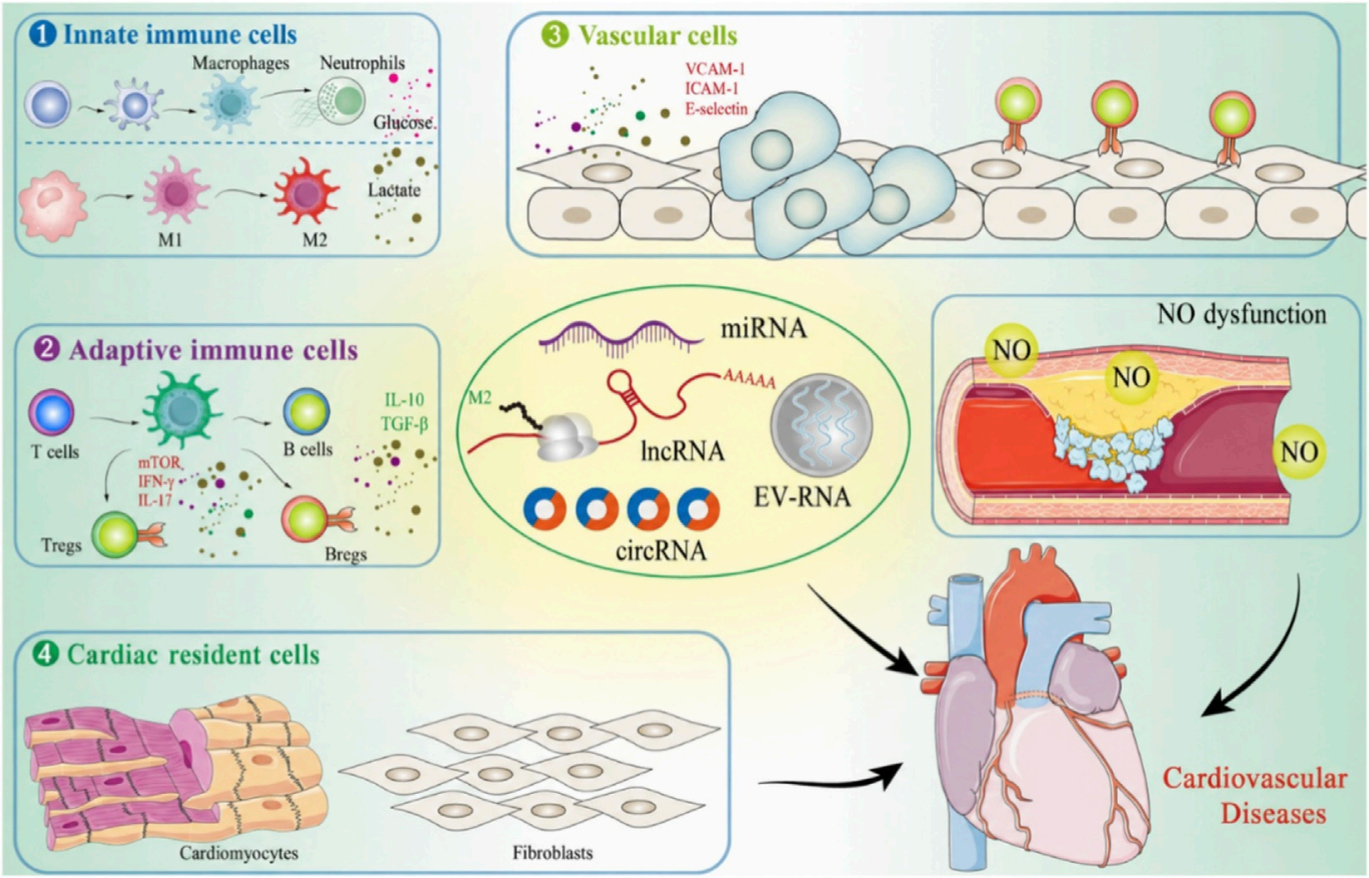
ncRNA-driven cardiovascular immunometabolic reprogramming. miRNAs, lncRNAs, circRNAs, and EV-associated RNAs regulate immune cells, endothelial cells, VSMCs, cardiomyocytes, and fibroblasts. These effects converge on cholesterol handling, inflammatory activation, endothelial dysfunction, mitochondrial stress, fibrosis, and adverse cardiovascular remodeling.

Non-coding RNAs reshape cardiovascular immunometabolism through layered regulation across immune, vascular, and cardiac resident cell compartments. Rather than acting as isolated disease markers, miRNAs, lncRNAs, circRNAs, and extracellular vesicle-associated ncRNAs modulate the metabolic state and inflammatory behavior of macrophages, monocytes, neutrophils, lymphocytes, endothelial cells, vascular smooth muscle cells, cardiomyocytes, and fibroblasts. These regulatory effects converge on several recurrent processes, including glycolytic activation, mitochondrial dysfunction, cholesterol handling, oxidative stress, inflammasome signaling, endothelial activation, fibrotic remodeling, and impaired inflammatory resolution. Importantly, the same ncRNA may have cell type-specific or context-dependent effects, explaining why findings in atherosclerosis, myocardial infarction, heart failure, and metabolic cardiomyopathy are sometimes apparently conflicting. A mechanistic interpretation of ncRNAs therefore requires attention to cellular source, disease stage, metabolic context, extracellular vesicle transfer, and species conservation (DeBerge et al., 2023; Thorp and Karlstaedt, 2024; Poller et al., 2018). Figure 2 summarizes the multilayered regulatory network through which ncRNAs coordinate immune-cell activation, vascular dysfunction, cardiomyocyte stress, fibroblast remodeling, and downstream cardiovascular disease progression.

### 3.1 Innate immune cells: macrophages, monocytes, and neutrophils

Macrophages and monocytes represent central cellular hubs in cardiovascular immunometabolism because their inflammatory phenotypes are tightly linked to glycolysis, mitochondrial respiration, cholesterol trafficking, fatty acid metabolism, and efferocytosis (Koelwyn et al., 2018; Groh et al., 2018). In atherosclerosis, plaque macrophages are exposed to modified lipoproteins, hypoxia, oxidative stress, apoptotic debris, and inflammatory cytokines, all of which reshape metabolic programs and reinforce inflammatory activation (Koelwyn et al., 2018; Tabas and Bornfeldt, 2020). miR-33 is one of the best-characterized ncRNA regulators of macrophage lipid metabolism, because anti-miR-33 therapy derepresses genes involved in mitochondrial respiration and ATP production while enhancing cholesterol efflux (Karunakaran et al., 2015). Genetic disruption of miR-33 targeting of ABCA1 further supports the view that miR-33 links cholesterol efflux to macrophage function and atherosclerotic plaque biology (Price et al., 2019). miR-155 provides a more complex example because it can promote inflammatory macrophage responses and atherogenesis by repressing Bcl6, whereas other studies suggest context-dependent anti-inflammatory feedback functions under specific experimental conditions (Nazari-Jahantigh et al., 2012; Li et al., 2016). This duality illustrates an important principle for this review: ncRNAs should not be interpreted as universally protective or pathogenic without defining cell type, stimulus, disease stage, and metabolic state (Nazari-Jahantigh et al., 2012; Li et al., 2016). miR-34a also contributes to macrophage immunometabolic dysfunction by regulating cholesterol efflux, inflammation, and atherosclerosis, suggesting that multiple miRNAs converge on macrophage lipid handling rather than acting through isolated pathways (Xu et al., 2020). LncRNAs extend this regulatory network beyond post-transcriptional repression, as shown by Chrome, a primate-enriched lncRNA elevated in plasma and plaques from individuals with coronary artery disease and involved in cholesterol homeostasis (Hennessy et al., 2019). MeXis also supports the importance of lncRNA-mediated transcriptional control in macrophage cholesterol efflux, linking lipid sensing to ABCA1-dependent anti-atherogenic programs (Sallam et al., 2018). Neutrophils should also be included in this mechanistic layer because neutrophil microvesicles can deliver miR-155 to disease-prone vascular regions and thereby amplify vascular inflammation and atherogenesis (Gomez et al., 2020). Together, these findings indicate that innate immune ncRNAs regulate not only cytokine expression but also the metabolic infrastructure that determines whether myeloid cells become lipid-loaded, inflammatory, reparative, or pro-resolving (Koelwyn et al., 2018; Tabas and Bornfeldt, 2020).

### 3.2 Adaptive immune cells and immune-metabolic balance

Compared with macrophages, the direct evidence linking ncRNAs to adaptive immune-cell immunometabolism in cardiovascular disease remains less developed, but this gap is itself important because T-cell and B-cell responses shape plaque inflammation, myocardial injury, and chronic remodeling (Baardman and Lutgens, 2020; Srikakulapu and McNamara, 2017). Regulatory T cells are metabolically distinct from effector T cells and participate in atherosclerotic immune control, suggesting that ncRNAs capable of altering Treg stability, lipid metabolism, or suppressive function could influence cardiovascular inflammatory endotypes (Baardman and Lutgens, 2020; Kuan et al., 2021). Experimental evidence that Tregs can license macrophage pro-resolving responses during regression of atherosclerosis further supports a cross-talk model in which adaptive immune metabolism indirectly reshapes myeloid ncRNA-regulated inflammatory programs (Sharma et al., 2020). miR-146a is relevant to this layer because it is associated with anti-inflammatory feedback, plaque stability, and NF-κB-related regulation in atherosclerotic contexts (Chu et al., 2022; Cheng et al., 2017). miR-155 is also relevant to adaptive immunity, but its cardiovascular interpretation is complicated by divergent effects across hematopoietic lineages, inflammatory stimuli, and experimental models (Ma et al., 2013; Tili et al., 2009). A critical limitation is that many cardiovascular ncRNA studies still measure mixed blood or plaque tissue rather than purified immune subsets, making it difficult to determine whether observed signals arise from T cells, B cells, monocytes, neutrophils, endothelial cells, or extracellular vesicles (Fasolo et al., 2019; Poller et al., 2018). Future work should therefore combine ncRNA profiling with single-cell immune phenotyping, metabolic inference, and functional perturbation to identify whether adaptive immune ncRNAs are causal drivers or secondary markers of cardiovascular inflammation (Fernandez et al., 2019; Han et al., 2023).

### 3.3 Vascular cells: endothelial cells and vascular smooth muscle cells

The lncRNA MALAT1 is also relevant to endothelial biology because it regulates endothelial cell function, vascular growth, and microvascular repair after myocardial infarction (Michalik et al., 2014; Chen et al., 2021). Hematopoietic MALAT1 deficiency can aggravate atherosclerotic lesion formation, indicating that MALAT1 may influence vascular disease through immune-cell as well as endothelial compartments (Cremer et al., 2019). MALAT1 is therefore not an endotype-specific single marker. Its interpretation differs when detected in endothelial cells as a vascular repair transcript, in hematopoietic or macrophage compartments as a plaque-inflammation modifier, or in mixed plasma/EV samples under metabolic stress. In Table 2, MALAT1 is therefore flagged as a context-dependent transcript rather than a unique vascular or metabolic classifier. Vascular smooth muscle cells are no longer viewed as passive structural cells, because they undergo phenotypic switching toward synthetic, inflammatory, macrophage-like, or osteochondrogenic states during atherosclerosis (Alencar et al., 2020). ncRNAs are highly relevant to this plasticity because miR-145 supports the contractile VSMC phenotype, whereas loss of contractile regulation contributes to plaque remodeling and vascular instability (Alencar et al., 2020; Cordes et al., 2009). CircRNAs are also emerging as regulators of VSMC behavior, although many reported circRNA-miRNA-mRNA axis mechanisms require additional experimental validation in disease-specific cardiovascular contexts (Wu et al., 2022; Lv et al., 2024). Thus, vascular ncRNAs couple immunometabolic stress to endothelial activation, defective repair, VSMC plasticity, and plaque remodeling (Fasolo et al., 2019; Schober et al., 2022).

### 3.4 Cardiac resident cells: cardiomyocytes and fibroblasts

Cardiomyocytes undergo profound metabolic remodeling during ischemia, hypertrophy, and heart failure, including altered mitochondrial respiration, substrate utilization, calcium handling, oxidative stress, and stress-responsive gene expression (DeBerge et al., 2023; Thorp and Karlstaedt, 2024). miR-210 is a representative hypoxia-responsive miRNA that regulates mitochondrial metabolism, bioenergetics, and reactive oxygen species flux during myocardial ischemia–reperfusion injury (Chan et al., 2009; Song et al., 2022). Cardiac-enriched lncRNAs also regulate maladaptive remodeling, as shown by Chast, which promotes cardiac remodeling, and Chaer, which functions as an epigenetic checkpoint for cardiac hypertrophy (Viereck et al., 2016; Wang et al., 2016). Cardiac fibroblasts form another essential layer because they integrate inflammatory cytokines, mechanical stress, TGF-β signaling, extracellular matrix remodeling, and paracrine communication after myocardial injury (van Rooij et al., 2008; Micheletti et al., 2017). miR-21 promotes cardiac fibroblast activation and fibrotic remodeling through pathways involving ERK–MAPK, STAT3, and TGF-β/Smad signaling (Thum et al., 2008; Zhou et al., 2018; Yuan et al., 2017). Macrophage-derived miR-21-containing particles can also contribute to pressure overload-induced cardiac fibrosis and dysfunction, highlighting the importance of immune-cell-to-fibroblast ncRNA communication (Ramanujam et al., 2021). By contrast, the miR-29 family is strongly linked to extracellular matrix regulation, and dysregulation of miR-29 after myocardial infarction supports its role as a regulator of cardiac fibrosis (van Rooij et al., 2008). The fibroblast-enriched lncRNA Wisper controls cardiac fibrosis and remodeling after injury, making it a strong example of cell-enriched lncRNA regulation with therapeutic implications (Micheletti et al., 2017). These examples show that cardiac ncRNAs regulate both intrinsic cardiomyocyte stress responses and intercellular inflammatory-fibrotic circuits that determine long-term remodeling (Caporali et al., 2024; Lu et al., 2022).

### 3.5 Circular RNAs: tissue-source signatures and endotype interpretation

CircRNAs should not be treated as a homogeneous ncRNA class, because their interpretation depends strongly on tissue source, disease stage, and cellular compartment. Cardiac or myocardial-enriched circRNA examples include circSLC8A1 and circNFIX, which have been proposed as auxiliary markers for acute ischemic heart disease-related sudden cardiac death, and MICRA, a blood-detectable myocardial infarction-associated circRNA linked to later left ventricular dysfunction (Tian et al., 2021; Vausort et al., 2016). CircFndc3b provides a mechanistic cardiac-repair example: it is downregulated after myocardial infarction and has been linked to endothelial repair, VEGF-A signaling, angiogenesis, and improved ventricular function in experimental models (Garikipati et al., 2019). These examples fit most naturally with the sterile ischemic injury and fibro-inflammatory remodeling axes, but they still require time-resolved human validation. Endothelial-enriched or endothelial-responsive circRNAs provide a second source pattern. cZNF292 is a hypoxia-regulated endothelial circRNA with proangiogenic activity, whereas circHIPK3 downregulation has been linked to high glucose-induced endothelial injury (Boeckel et al., 2015; Cao et al., 2018). These transcripts are therefore more plausibly aligned with vascular immune-endothelial dysfunction and metabolic-inflammatory vascular stress than with a generic cardiovascular disease label. Immune-cell-enriched or immune-active circRNA evidence is also emerging. CircANRIL, derived from the 9p21 atherosclerosis locus, modulates ribosomal RNA maturation, nucleolar stress, and atheroprotection in human vascular disease-relevant cells, while macrophage-specific circHIPK2 has recently been linked to post-myocardial-infarction inflammation and fibrosis through stress-granule and inflammatory signaling pathways (Holdt et al., 2016; Jung et al., 2026). Thus, athero-inflammatory and fibro-inflammatory endotypes may show distinct circRNA source signatures only when blood or tissue data are deconvoluted by cell type. At present, however, no patient-level circRNA tissue-source signature has been prospectively validated to assign cardiovascular endotypes. Therefore, circSLC8A1, circNFIX, MICRA, circFndc3b, cZNF292, circHIPK3, circANRIL, and circHIPK2 should be interpreted as mechanistic or biomarker candidates rather than established classifiers. Future work should combine cell-sorted RNA sequencing, single-cell or spatial methods, EV-source deconvolution, and longitudinal sampling to test whether cardiac-, immune-, endothelial-, and vascular-derived circRNAs distinguish endotypes beyond conventional biomarkers.

### 3.6 Fibro-inflammatory remodeling in HFrEF and HFpEF

The fibro-inflammatory remodeling endotype deserves explicit treatment because it is central to HFrEF and also contributes to HFpEF, aortic stenosis, pressure overload, and post-infarction ventricular remodeling. Mechanistically, this endotype is not simply collagen accumulation. It reflects persistent cytokine signaling, macrophage-fibroblast communication, myofibroblast expansion, extracellular matrix cross-linking, and failed resolution of repair. miR-21 promotes fibroblast activation and fibrotic remodeling through ERK-MAPK, STAT3, and TGF-beta/Smad-related pathways, and macrophage-derived miR-21-containing particles can transmit pressure-overload signals to fibroblasts (Thum et al., 2008; Zhou et al., 2018; Yuan et al., 2017; Ramanujam et al., 2021). In contrast, the miR-29 family represses extracellular matrix genes and is typically interpreted as an anti-fibrotic regulatory node after myocardial injury (van Rooij et al., 2008). Wisper adds a cell-enriched lncRNA example because it is enriched in cardiac fibroblasts and controls cardiac fibrosis and remodeling after injury (Micheletti et al., 2017).

Recent evidence further links cytokine-driven fibrosis to ncRNA outputs. Tikhomirov et al. identified IL-11-induced miR-27b-5p and miR-497-5p as downstream functional mediators and circulating extracellular-vesicle-associated indicators of cardiac fibrosis, thereby connecting a pro-fibrotic cytokine axis with measurable miRNA changes (Tikhomirov et al., 2026). Boichenko et al. also emphasized that circulating miRNAs, lncRNAs, and circRNAs may indicate fibrosis and heart failure severity across HFrEF and HFpEF, reinforcing the point that ejection fraction categories do not map cleanly onto molecular remodeling states (Boichenko et al., 2025). In an endotype workflow, fibro-inflammatory remodeling should therefore be assigned using concordant evidence from ejection fraction, fibrosis imaging such as CMR extracellular volume or late gadolinium enhancement, extracellular matrix biomarkers, IL-11/TGF-beta-related signaling, and candidate ncRNAs rather than by miR-21 or miR-29 expression alone.

### 3.7 Extracellular vesicles and ncRNA-metabolite-epigenetic feedback loops

**FIGURE 3:**
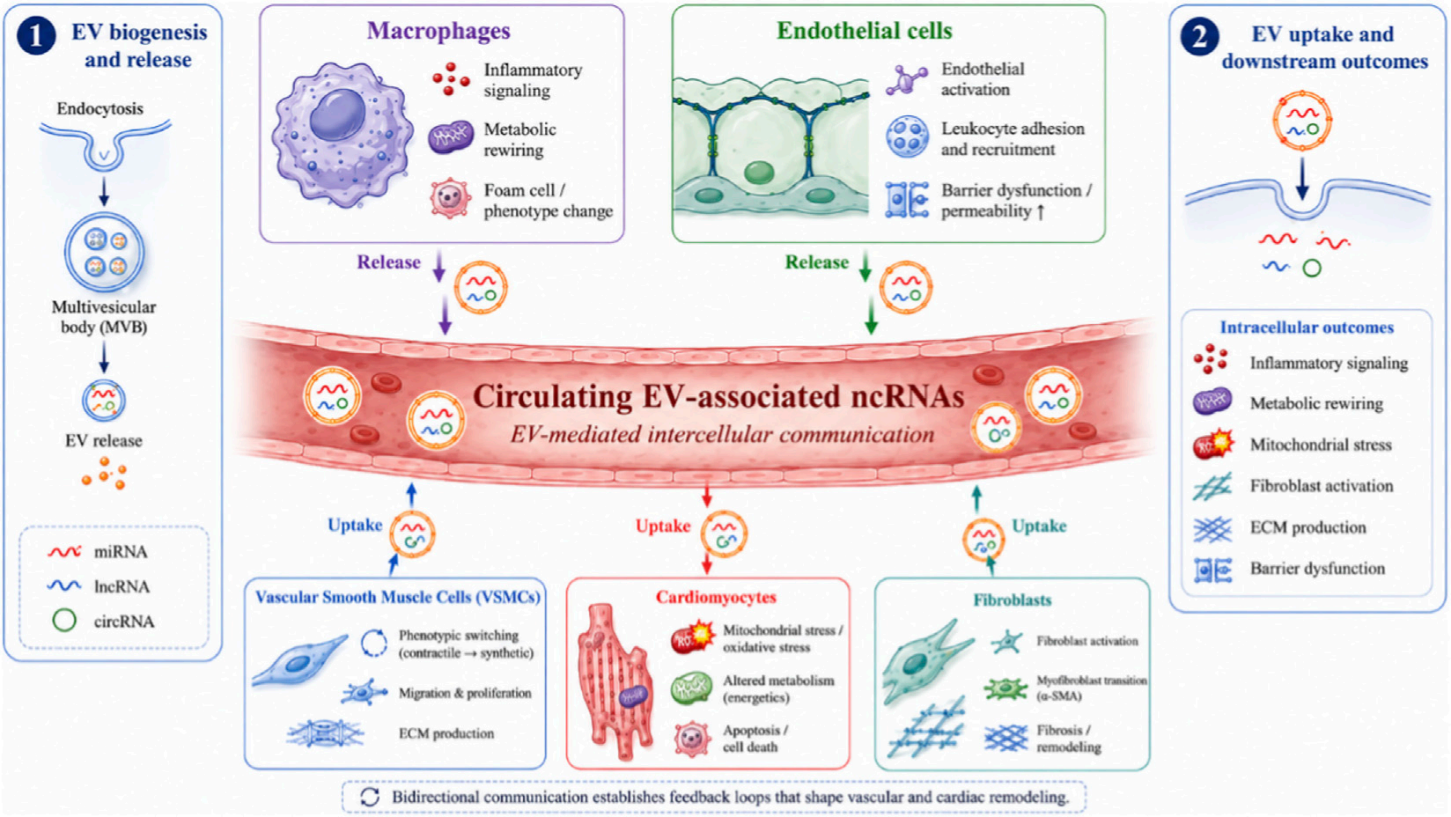
EV-associated ncRNA-mediated cardiovascular cell-cell communication. EVs released by immune, vascular, and cardiac resident cells transport miRNAs, lncRNAs, and circRNAs to recipient cells. This transfer can propagate inflammation, metabolic rewiring, mitochondrial stress, endothelial dysfunction, vascular remodeling, fibroblast activation, ECM deposition, and angiogenic imbalance.

Extracellular vesicles provide a mechanism by which ncRNAs move between cardiomyocytes, endothelial cells, immune cells, fibroblasts, and vascular smooth muscle cells, thereby converting local cellular stress into tissue-level communication (Das and Halushka, 2015; Jiapaer et al., 2023). Figure 3 highlights EV-associated ncRNAs as mobile intercellular messengers that transmit inflammatory, metabolic, mitochondrial, endothelial, and fibrotic signals among immune, vascular, and cardiac resident cells. EV-associated ncRNAs are particularly important for cardiovascular immunometabolism because EVs may influence immune-cell metabolic states during cardiac injury and obesity-associated cardiovascular stress (Omoto et al., 2024). In MI-related and chronic heart failure models, EV-enriched miR-27a-5p, reported in the original study as miR-27a*, has been linked to cardiac hypertrophic remodeling and impaired antioxidant-defense signaling, indicating that extracellular miRNAs may connect oxidative stress with post-injury or chronic remodeling (Tian et al., 2020). More broadly, extracellular ncRNAs may serve as disease mediators, biomarkers, and therapeutic cargos, but their interpretation requires careful distinction between passive release from injured cells and active vesicle-mediated signaling (Jiapaer et al., 2023; Zhao et al., 2022). A further mechanistic layer involves reciprocal feedback between ncRNAs, metabolites, and epigenetic or epitranscriptomic regulation (Kumari et al., 2022; Qin et al., 2020). m6A RNA methylation is increasingly recognized in cardiovascular disease and may affect inflammation, hypertrophy, ischemic injury, and heart failure through altered RNA stability, translation, and regulatory RNA function (Qin et al., 2020; Wang et al., 2023). Metabolites such as lactate, succinate, acetyl-CoA, NAD^+^, and reactive oxygen species can alter chromatin and RNA regulatory states, while ncRNAs can reciprocally control metabolic enzymes, transporters, mitochondrial regulators, and inflammatory pathways (DeBerge et al., 2023; Thorp and Karlstaedt, 2024). This feedback-loop model is especially valuable for interpreting chronic cardiovascular inflammation, where transient metabolic stress may become stabilized as inflammatory memory, fibrotic remodeling, or persistent endothelial dysfunction (DeBerge et al., 2023; Kumari et al., 2022). Therefore, the most informative future studies will not merely identify differentially expressed ncRNAs but will map ncRNA function within defined cell types, metabolic states, extracellular communication routes, and epigenetic regulatory circuits (Fernandez et al., 2019; Han et al., 2023). As summarized in Table 4, representative miRNAs, lncRNAs, circRNAs, and EV-associated ncRNAs regulate cardiovascular immunometabolic reprogramming across immune, vascular, and cardiac resident-cell compartments, thereby providing both mechanistic targets and translational entry points.

**TABLE 4:** Representative ncRNA regulators in cardiovascular immunometabolic reprogramming.

ncRNA or class	Major cellular context	Main immunometabolic function	Cardiovascular relevance	Refs
miR-33	Macrophages and atherosclerotic plaques	Regulates cholesterol efflux, mitochondrial respiration, ATP production, and macrophage lipid metabolism	Links foam-cell formation to athero-inflammatory plaque progression	Karunakaran et al. (2015); Price et al. (2019)
miR-155	Macrophages, endothelial cells, VSMCs, and neutrophil-derived vesicles	Modulates inflammatory activation and vascular immune responses in a context-dependent manner	Illustrates cell type- and stage-dependent ncRNA effects in vascular inflammation	Nazari-Jahantigh et al. (2012); Li et al. (2016); Gomez et al. (2020)
miR-34a	Macrophages	Regulates cholesterol efflux, inflammatory signaling, and macrophage dysfunction	Contributes to macrophage lipid imbalance and atherosclerotic inflammation	Xu et al. (2020)
CHROME and MeXis	Macrophages and lipid-regulating compartments	Control cholesterol homeostasis and ABCA1-dependent cholesterol efflux	Connect lipid sensing with anti-atherogenic macrophage programs	Hennessy et al. (2019); Sallam et al. (2018)
miR-146a	Immune cells and atherosclerotic lesions	Participates in NF-kappaB-related anti-inflammatory feedback	May contribute to plaque stability and inflammatory restraint	Chu et al. (2022); Cheng et al. (2017)
miR-126-5p	Endothelial cells	Maintains endothelial proliferative reserve and vascular homeostasis	Supports endothelial repair and influences atherosclerotic lesion formation	Schober et al. (2014)
miR-92a	Endothelial cells and ischemic tissues	Limits angiogenesis and endothelial recovery	Inhibition may enhance neovascularization and vascular repair after ischemic injury	Bonauer et al. (2009); Daniel et al. (2014)
miR-145	Vascular smooth muscle cells	Maintains the contractile VSMC phenotype and restrains phenotypic switching	Relevant to vascular remodeling, plaque stability, and VSMC plasticity	Cordes et al. (2009); Chin et al. (2021)
MALAT1	Endothelial and hematopoietic compartments	Regulates endothelial function, vascular growth, and inflammation-associated vascular remodeling	Shows context-dependent roles in myocardial repair and atherosclerosis	Michalik et al. (2014); Cremer et al. (2019)
miR-210	Cardiomyocytes under hypoxic or ischemic stress	Regulates mitochondrial metabolism, bioenergetics, and ROS production	Links hypoxia-responsive ncRNA regulation to ischemia-reperfusion injury	Chan et al. (2009); Song et al. (2022)
miR-21, miR-29, and Wisper	Cardiac fibroblasts and macrophage-fibroblast circuits	Regulate fibroblast activation, ECM remodeling, TGF-beta signaling, and fibrosis	Provide anti-fibrotic candidate nodes for adverse cardiac remodeling	van Rooij et al. (2008); Micheletti et al. (2017); Thum et al. (2008)
EV-associated ncRNAs	Cardiomyocytes, endothelial cells, immune cells, fibroblasts, VSMCs, and platelets	Mediate intercellular RNA transfer and tissue-level stress communication	Function as biomarkers, mediators, and potential therapeutic cargos	Jiapaer et al. (2023); Omoto et al. (2024); Zhao et al. (2022)
Representative circRNAs (circSLC8A1/circNFIX, MICRA, circFndc3b, cZNF292, circANRIL, circHIPK2)	Cardiomyocytes/myocardial tissue, blood after MI, endothelial cells, VSMCs, and macrophages	Reflect or regulate ischemic injury, endothelial repair, angiogenesis, ribosomal stress, macrophage inflammation, and fibrotic remodeling	Provide tissue-source-specific candidates for ischemic, vascular, athero-inflammatory, and fibro-inflammatory endotype hypotheses	Tian et al. (2021); Vausort et al. (2016); Garikipati et al. (2019); Boeckel et al. (2015); Holdt et al. (2016); Jung et al. (2026)

## 4 Therapeutic opportunities: from ncRNA biomarkers to endotype-guided intervention

The therapeutic value of cardiovascular ncRNA biology should be considered at three interconnected levels: biomarker development, mechanism-guided target discovery, and RNA-based intervention. Circulating miRNAs, lncRNAs, circRNAs, and extracellular vesicle-associated ncRNAs may help identify candidate inflammatory endotypes, monitor disease progression, and predict therapeutic responsiveness. At the mechanistic level, ncRNAs provide entry points for modulating macrophage lipid handling, endothelial activation, cardiomyocyte stress, fibroblast remodeling, mitochondrial dysfunction, and immune-metabolic polarization. At the therapeutic level, antisense oligonucleotides, antagomiRs, miRNA mimics, siRNAs, and engineered extracellular vesicles offer attractive but technically demanding strategies. The field has already entered clinical translation, as illustrated by inclisiran for PCSK9 silencing and CDR132L for miR-132 inhibition in heart failure. However, the phase 2 CDR132L experience also shows that pharmacodynamic target engagement and tolerability do not automatically translate into significant structural or clinical efficacy, underscoring the need for rigorous endotype selection, tissue-specific delivery, long-term safety evaluation, and validation beyond small discovery cohorts (Ardiana et al., 2023; Shah and Giacca, 2022; Ray et al., 2020; Taubel et al., 2021; Bauersachs et al., 2026).

### 4.1 Circulating and extracellular vesicle-associated ncRNAs as liquid biopsy tools

Circulating ncRNAs are attractive cardiovascular biomarkers because they can be detected in serum, plasma, whole blood, platelets, or extracellular vesicles and may reflect injury, inflammation, vascular dysfunction, or remodeling across multiple tissue compartments (Poller et al., 2018; Viereck and Thum, 2017). Compared with conventional markers such as troponin, natriuretic peptides, LDL-C, and high-sensitivity C-reactive protein, ncRNA profiles may capture upstream regulatory programs rather than only downstream injury or inflammatory burden (Poller et al., 2018; Viereck and Thum, 2017). This distinction is important for the present review because inflammatory endotypes are defined by dominant mechanisms, and ncRNAs may help distinguish macrophage lipid-overload signatures, endothelial immune activation, ischemia-related sterile inflammation, metabolic-inflammatory HFpEF, or fibro-inflammatory remodeling (Caporali et al., 2024; de Gonzalo-Calvo et al., 2019a). Extracellular vesicle-associated ncRNAs are particularly relevant because EVs protect RNA cargoes from degradation and may provide information about cell-to-cell communication among cardiomyocytes, endothelial cells, fibroblasts, vascular smooth muscle cells, and immune cells (Poller et al., 2018; Jiapaer et al., 2023). EV-ncRNAs may therefore function as both biomarkers and biological mediators, which makes them more mechanistically informative than freely circulating transcripts but also more difficult to standardize analytically (Jiapaer et al., 2023; Zhao et al., 2022). For clinical use, single ncRNA markers are unlikely to be sufficient because cardiovascular diseases are heterogeneous, comorbidity-rich, and strongly influenced by age, sex, medication exposure, renal function, metabolic status, and sampling conditions (Poller et al., 2018; Viereck and Thum, 2017). A more realistic strategy is to develop multi-ncRNA panels integrated with clinical variables, imaging parameters, inflammatory biomarkers, lipid profiles, and metabolomic signatures (Caporali et al., 2024; de Gonzalo-Calvo et al., 2019a). However, many published ncRNA biomarker studies remain limited by small sample size, inconsistent normalization methods, different biospecimen types, variable pre-analytical handling, and insufficient external validation (Poller et al., 2018; Viereck and Thum, 2017). Thus, ncRNA-based liquid biopsy should be positioned not as an immediate replacement for established cardiovascular biomarkers but as a complementary tool for mechanism-based stratification and therapeutic decision support (Caporali et al., 2024; de Gonzalo-Calvo et al., 2019a).

### 4.2 ncRNA-targeted therapeutics: current modalities and cardiovascular examples

Therapeutic modulation of ncRNAs can be achieved through several platforms, including antisense oligonucleotides, locked nucleic acid inhibitors, antagomiRs, miRNA mimics, siRNAs, small activating RNAs, and RNA-loaded nanoparticles or extracellular vesicles (Ardiana et al., 2023; Shah and Giacca, 2022). The strongest clinical proof of concept for RNA interference in cardiovascular medicine is inclisiran, a chemically modified siRNA that targets hepatic PCSK9 mRNA and produces sustained LDL-C lowering with twice-yearly maintenance dosing after initial administration (Ray et al., 2020; Wright et al., 2021). Although inclisiran targets a protein-coding transcript rather than an endogenous ncRNA, it demonstrates that durable RNA-based silencing can be clinically feasible in cardiovascular risk reduction (Ray et al., 2020). For ncRNA-directed therapy, CDR132L remains an important example because it targets miR-132 and first showed tolerability, linear pharmacokinetics, and durable pharmacodynamic target engagement in a first-in-human phase 1b randomized study in patients with heart failure (Taubel et al., 2021). New phase 2 data now require a more cautious interpretation. In the randomized HF-REVERT trial (Bauersachs et al., 2024), 294 patients with recent myocardial infarction and left ventricular systolic dysfunction were assigned to CDR132L 5 mg/kg, CDR132L 10 mg/kg, or placebo, and 280 treated patients were included in the modified intention-to-treat analysis (Bauersachs et al., 2026). CDR132L was well tolerated and produced dose-dependent plasma miR-132 suppression, but the primary endpoint, percentage change in left ventricular end-systolic volume index at 6 months, did not differ significantly between either CDR132L dose and placebo; secondary endpoints including left ventricular ejection fraction, global longitudinal strain, and NT-proBNP were also not significantly different (Bauersachs et al., 2026). Exploratory signals in patients with advanced remodeling should therefore be viewed as hypothesis-generating rather than proof of clinical efficacy. Thus, CDR132L should be interpreted as evidence that cardiovascular anti-miRNA therapy can achieve target engagement and acceptable short-term safety, while also illustrating the need for disease-stage- and remodeling-enriched trials. miR-92a inhibition provides another translationally relevant example because antagomir-mediated suppression of miR-92a improved angiogenesis and functional recovery in ischemic models, including limb ischemia and myocardial infarction (Bonauer et al., 2009; Hinkel et al., 2013). Targeting miR-21 has also shown therapeutic potential in fibrotic remodeling, and antimiR-21 treatment prevented myocardial dysfunction in a pig model of ischemia/reperfusion injury, supporting the value of large-animal validation before clinical translation (Hinkel et al., 2020). LncRNA-targeted therapy remains less mature but is conceptually attractive because disease-associated lncRNAs can show tissue-enriched or cell-enriched expression patterns (Ardiana et al., 2023; Haemmig and Feinberg, 2017). Wisper is an instructive example because it is enriched in cardiac fibroblasts and regulates cardiac fibrosis and remodeling after injury, suggesting that fibroblast-associated lncRNAs may offer more cell-selective therapeutic entry points than broadly expressed miRNAs (Micheletti et al., 2017). Nevertheless, lncRNA therapeutics face major barriers, including poor sequence conservation between species, incomplete annotation, complex subcellular localization, and uncertain mechanisms of action (Ardiana et al., 2023; Haemmig and Feinberg, 2017).

### 4.3 Endotype-guided intervention: matching ncRNA targets to dominant mechanisms

The most important therapeutic implication of this review is that ncRNA interventions should be matched to research-defined inflammatory endotypes rather than applied uniformly across broad disease categories (Caporali et al., 2024; de Gonzalo-Calvo et al., 2019a). In an athero-inflammatory endotype, rational ncRNA targets would include regulators of macrophage cholesterol efflux, foam cell formation, inflammasome activation, endothelial adhesion, and vascular smooth muscle cell plasticity (Schober et al., 2022; Hennessy et al., 2019; Sallam et al., 2018). In this setting, ncRNA therapy might be combined with LDL-C lowering, anti-inflammatory treatment, or plaque-stabilizing strategies to reduce residual inflammatory risk that persists despite lipid control (Ridker et al., 2017; Ridker et al., 2020). In an ischemia–reperfusion inflammatory endotype, candidate ncRNA interventions should focus on mitochondrial protection, oxidative stress reduction, cardiomyocyte survival, neutrophil recruitment, macrophage transition, and inflammatory resolution (DeBerge et al., 2023; Song et al., 2022). In a fibro-inflammatory remodeling endotype, therapeutic priority should shift toward fibroblast activation, extracellular matrix deposition, TGF-β signaling, macrophage–fibroblast communication, and adverse ventricular remodeling (Micheletti et al., 2017; Hinkel et al., 2020). In a metabolic-inflammatory endotype, particularly in obesity, diabetes, and HFpEF, ncRNA targets should be evaluated for their ability to regulate substrate utilization, endothelial metabolism, adipose–cardiac inflammation, mitochondrial function, and immune-cell metabolic polarization (Thorp and Karlstaedt, 2024; Schiattarella et al., 2021; Sabbah et al., 2020). This endotype-guided model may also improve biomarker development because the relevant ncRNA panel for macrophage-rich atherosclerosis is unlikely to be identical to that for HFpEF, post-infarction remodeling, or hypertensive vascular dysfunction (Caporali et al., 2024; de Gonzalo-Calvo et al., 2019a). Therefore, future trials should avoid enrolling biologically mixed populations without mechanistic stratification or fibrosis/remodeling enrichment when relevant, because such designs may dilute treatment effects and obscure responder subgroups (Caporali et al., 2024; de Gonzalo-Calvo et al., 2019a).

### 4.4 Multicomponent and traditional medicine-inspired strategies

Beyond single-target RNA therapeutics, multicomponent intervention represents a possible but still preliminary strategy for modulating ncRNA-driven cardiovascular immunometabolism. This perspective is relevant to Traditional Chinese Medicine because many formulations contain multiple bioactive compounds and may influence interconnected inflammatory, oxidative, metabolic, and fibrotic pathways (Lin et al., 2020). However, evidence that these interventions act through ncRNA-dependent mechanisms remains largely hypothesis-generating unless supported by target-engagement assays, gain- and loss-of-function experiments, pharmacokinetic data, dose-response studies, and clinically relevant disease models (Wang et al., 2024; Liu et al., 2023). Network pharmacology predictions or descriptive expression changes alone are insufficient to establish ncRNAs as causal mediators. A more rigorous strategy is to use ncRNAs as predefined mechanistic verification nodes. For example, candidate interventions for a fibro-inflammatory remodeling endotype could be tested for effects on miR-21-, miR-29-, or lncRNA-associated fibrotic programs, whereas interventions for an athero-inflammatory endotype should be evaluated for effects on macrophage cholesterol efflux, inflammasome activation, endothelial adhesion, and plaque inflammation. Clinical translation should also be endotype-stratified. The QUEST trial of Qiliqiangxin in heart failure with reduced ejection fraction shows that selected Traditional Chinese Medicine formulations can be assessed in randomized cardiovascular outcome trials, but whether the observed benefit involves ncRNA-dependent immunometabolic reprogramming remains unresolved and requires biomarker-rich mechanistic studies (Cheang et al., 2024).

### 4.5 Translational bottlenecks and safety considerations

Despite strong biological rationale, ncRNA therapeutics face substantial translational barriers in cardiovascular disease (Ardiana et al., 2023; Shah and Giacca, 2022). The first barrier is delivery, because efficient and selective targeting of myocardium, vascular wall, plaque macrophages, endothelial cells, or cardiac fibroblasts is more difficult than hepatic delivery (Ardiana et al., 2023; Shah and Giacca, 2022). The second barrier is specificity, because a single miRNA can regulate many transcripts and may exert different effects in cardiomyocytes, endothelial cells, macrophages, fibroblasts, and immune cells (Caporali et al., 2024; de Gonzalo-Calvo et al., 2019a). The third barrier is disease-stage dependency, since inhibiting an inflammatory ncRNA may be beneficial during chronic remodeling but harmful during early repair or inflammatory resolution (DeBerge et al., 2023; Caporali et al., 2024). The fourth barrier is species translation, which is particularly problematic for lncRNAs and circRNAs because many are poorly conserved or incompletely annotated across humans and experimental animals (Ardiana et al., 2023; Haemmig and Feinberg, 2017). The fifth barrier is safety, including immune activation by oligonucleotides, off-target effects, unintended gene-network perturbation, hepatic or renal accumulation, and uncertainty regarding long-term suppression of regulatory RNAs (Ardiana et al., 2023; Shah and Giacca, 2022). These challenges mean that successful ncRNA therapy will require chemically optimized molecules, validated delivery systems, tissue-specific targeting strategies, robust pharmacodynamic biomarkers, and careful patient selection (Ardiana et al., 2023; Shah and Giacca, 2022).

### 4.6 Practical roadmap for clinical translation

A practical roadmap should begin with discovery cohorts that define ncRNA signatures within carefully phenotyped inflammatory endotypes rather than within broad diagnostic labels (Caporali et al., 2024; de Gonzalo-Calvo et al., 2019a). Candidate ncRNAs should then be validated in independent human cohorts, mapped to specific cell types using single-cell and spatial approaches, and tested functionally through gain- and loss-of-function models (Han et al., 2023; de Gonzalo-Calvo et al., 2019a). Mechanistic studies should include metabolic flux analysis, mitochondrial assays, inflammatory pathway readouts, extracellular vesicle tracking, and cell-specific perturbation to confirm that the ncRNA participates in immunometabolic reprogramming rather than simply reflecting tissue injury (DeBerge et al., 2023; Thorp and Karlstaedt, 2024). Therapeutic candidates should then advance through pharmacokinetic, biodistribution, target-engagement, toxicity, and large-animal efficacy studies before entering endotype-enriched clinical trials (Taubel et al., 2021; Hinkel et al., 2020). Ultimately, the most promising application may be a combined strategy in which ncRNA biomarkers classify inflammatory endotypes, ncRNA therapeutics modulate causal regulatory nodes, and conventional cardiovascular drugs target downstream risk factors and organ-level dysfunction (Caporali et al., 2024; Shah and Giacca, 2022).

## 5 Limitations of this review

This review has several limitations. First, it is a narrative synthesis rather than a systematic review or meta-analysis; therefore, the cited examples were selected for mechanistic relevance, endotype representation, and translational value rather than through quantitative evidence pooling. Second, the endotype framework remains a conceptual summary and research-grade assignment scheme, not a clinically validated diagnostic algorithm. No universally accepted thresholds currently define the proposed ncRNA-based cardiovascular endotypes, and the proposed categories may overlap within individual patients, particularly in advanced atherosclerosis, ischemic heart failure, metabolic syndrome, and HFpEF. Third, many ncRNA findings are derived from animal models, mixed tissues, or small human cohorts, and the cellular source, temporal dynamics, and causal role of circulating ncRNAs often remain uncertain.

Fourth, therapeutic translation is constrained by tissue-selective delivery, off-target gene-network effects, immune activation, species conservation, disease-stage dependency, and long-term safety. These limitations are especially important for lncRNAs and circRNAs, which are often less conserved and less completely annotated than miRNAs. Fifth, negative or neutral clinical readouts, such as the phase 2 CDR132L trial, should be incorporated into the evidentiary framework rather than treated as exceptions, because they clarify the gap between target engagement and clinical efficacy. Finally, traditional medicine-inspired ncRNA studies require particular caution because many current reports are based on predicted networks or limited validation. These issues should be addressed by integrating standardized biospecimen handling, independent validation cohorts, single-cell and spatial mapping, functional perturbation, pharmacokinetic assessment, and endotype-enriched clinical designs.

## 6 Conclusion and outlook

**FIGURE 4:**
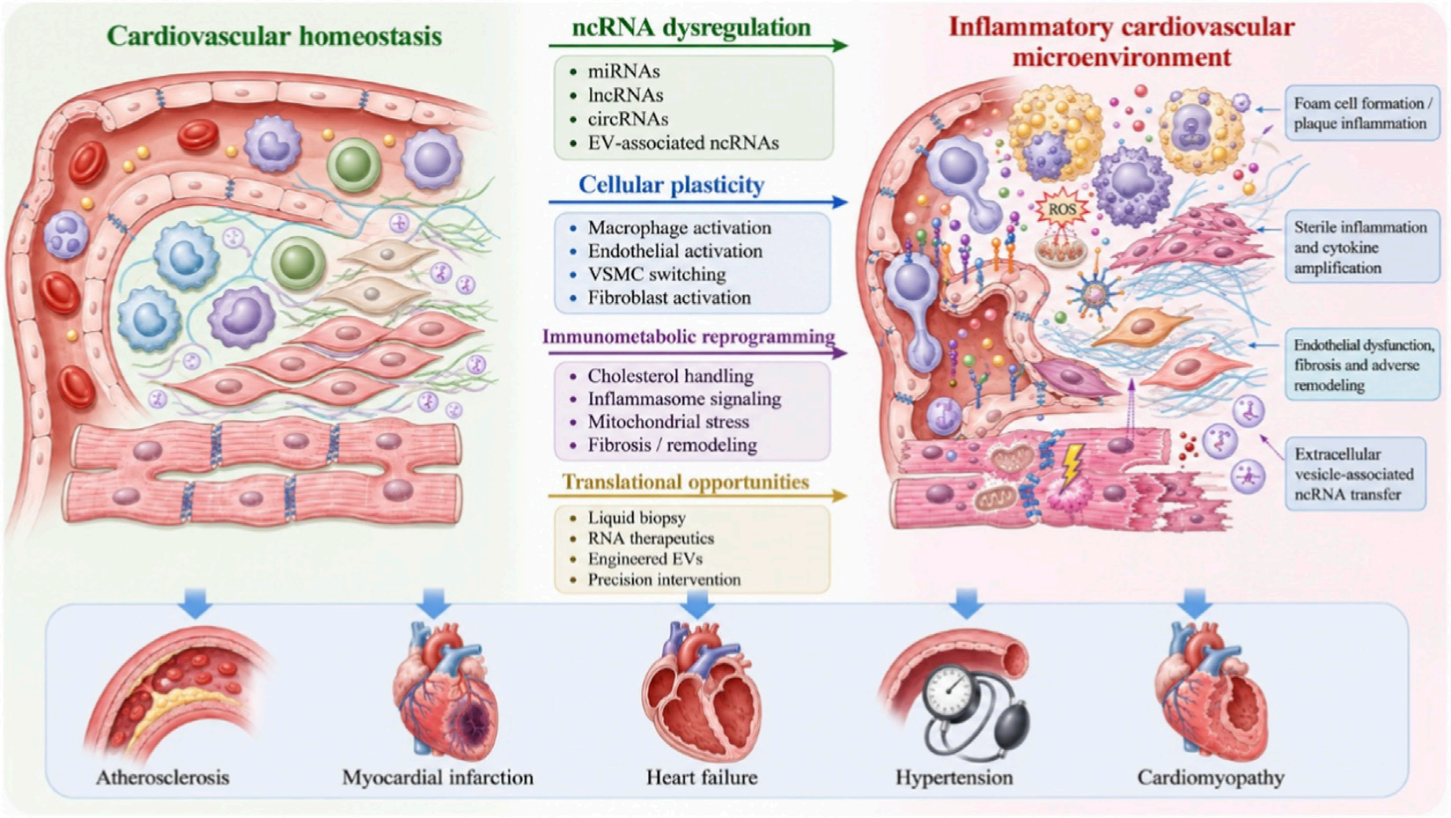
Integrated overview of ncRNA-driven cardiovascular immunometabolic reprogramming. ncRNA dysregulation links loss of cardiovascular homeostasis to cellular plasticity, inflammatory microenvironment formation, fibrosis, and major cardiovascular phenotypes. The model also highlights translational opportunities involving liquid biopsy, RNA therapeutics, engineered EVs, and precision intervention strategies.

Non-coding RNAs provide a mechanistic link between inflammation, metabolism, extracellular communication, and cardiovascular remodeling (Figure 4). The inflammatory endotype framework proposed here is intended to make this biology more research-useful by connecting ncRNA signals with dominant pathogenic mechanisms, relevant sample types, evidence strength, and therapeutic opportunities, while recognizing that it is not yet a validated diagnostic system. Three actionable messages emerge. First, ncRNA profiling should be interpreted within defined inflammatory and immunometabolic contexts rather than broad diagnostic labels alone. Second, candidate ncRNAs should be advanced only when circulating signals can be linked to cellular source, disease stage, and functional mechanism. Third, future therapeutic development should combine endotype-enriched patient selection, tissue-specific delivery, pharmacodynamic biomarkers, and rigorous safety evaluation. In this way, ncRNAs may serve not only as biomarkers but also as candidate classifiers, regulatory nodes, and therapeutic targets for precision cardiovascular medicine.
